# Solitary fibrous tumor of the gallbladder: a case report

**DOI:** 10.1186/s40792-024-02057-8

**Published:** 2024-11-18

**Authors:** Kiwako Sekine, Yuichi Nakaseko, Keigo Nakashima, Teppei Kamada, Junji Takahashi, Manabu Koja, Naoko Fukushima, Ryota Iwase, Teruyuki Usuba, Masaichi Ogawa, Yutaka Suzuki

**Affiliations:** 1 Department of Surgery, International University of Health and Welfare Hospital, 537-3, Iguchi, Nasushiobara, Tochigi 329-2763 Japan; 2 Department of Surgery, The Jikei University Katsushika Medical Center, 6-41-2, Aoto, Katsushika-Ku, Tokyo, 125-8506 Japan

**Keywords:** Case report, Solitary fibrous tumor, Gallbladder

## Abstract

**Background:**

Primary solitary fibrous tumors (SFTs) of the gallbladder are rare. Here, we report the case of a patient who underwent surgical treatment for a primary SFT originating in the gallbladder.

**Case presentation:**

A 48-mm gallbladder tumor was detected in a 70-year-old man using abdominal ultrasonography at a primary hospital, and he was subsequently referred to our department. A 50-mm enhanced tumor in the gallbladder was identified using computed tomography. Magnetic resonance imaging revealed a smooth-marginated tumor with hyperintensity on T2-weighted imaging. 18F-Fluorodeoxyglucose positron emission tomography confirmed high-level fluorodeoxyglucose uptake in the gallbladder tumor in the early phase without increasing uptake in the later phase. Surgical resection was planned to evaluate the tumor diagnosis. Initially, we performed open cholecystectomy with wedge resection of the gallbladder bed. Intraoperative pathological examination suggested gallbladder cancer; therefore, we performed radical surgery, including resection of the common bile duct, extended radical lymphadenectomy, and choledochojejunostomy. Ultimately, the final pathological examination revealed an SFT originating from the gallbladder with a negative surgical margin. Postoperatively, the patient developed bile leakage that was treated with tube drainage. The patient recovered satisfactorily and was discharged on postoperative day 20. At 24 months postoperatively, the patient was in good general condition without recurrence.

**Conclusions:**

We report a rare case of a primary SFT originating in the gallbladder. Clinicians should be aware that SFT can be found in the gallbladder, and when it is difficult to make a preoperative diagnosis, surgical treatment should be considered.

## Background

Solitary fibrous tumors (SFTs) are relatively rare, borderline, malignant soft tissue tumors that originate from mesenchymal cells. Surgical resection is the therapeutic strategy for SFT, and most patients have a good prognosis after surgery.

SFTs can occur in any part of the body, but they most commonly arise in the intrathoracic region, pleura, or lungs. The second most common site is the abdominal cavity [1]; however, primary SFTs rarely occur in the gallbladder. When we searched the PubMed database for publications containing the terms “solitary fibrous tumor” and “gallbladder”, there was only one report of primary SFT occurring in the gallbladder that was detected incidentally in the resected specimen [2]. Therefore, it would have been challenging to consider that a tumor in the gallbladder could be an SFT on preoperative images.

Herein, we report a case of an SFT of the gallbladder that was detected preoperatively and was successfully treated surgically.

## Case presentation

**Fig. 1:**
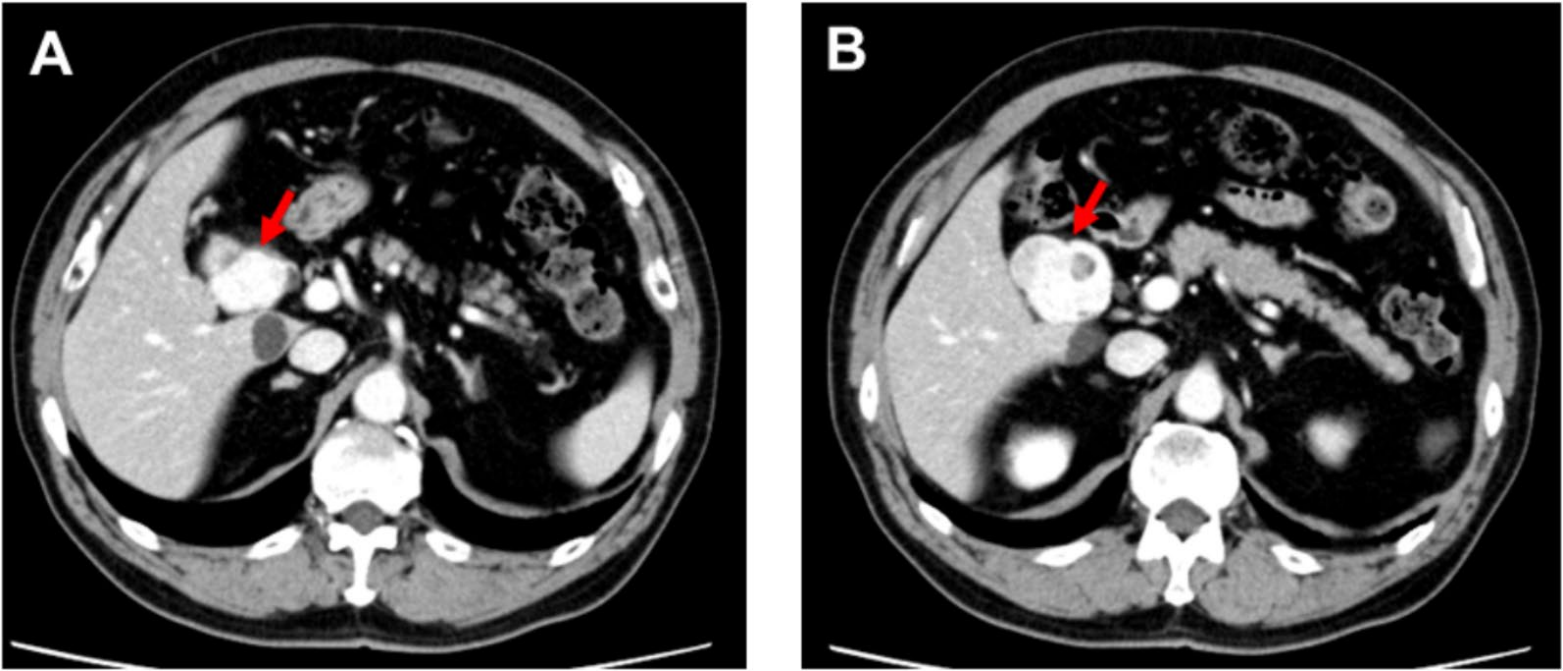
Abdominal contrast-enhanced computed tomography imaging. **A** A mass measuring 48 mm in diameter on the right side of the common bile duct (arrow). **B** The border of the mass was clear with no obvious invasion (arrow)

**Fig. 2:**
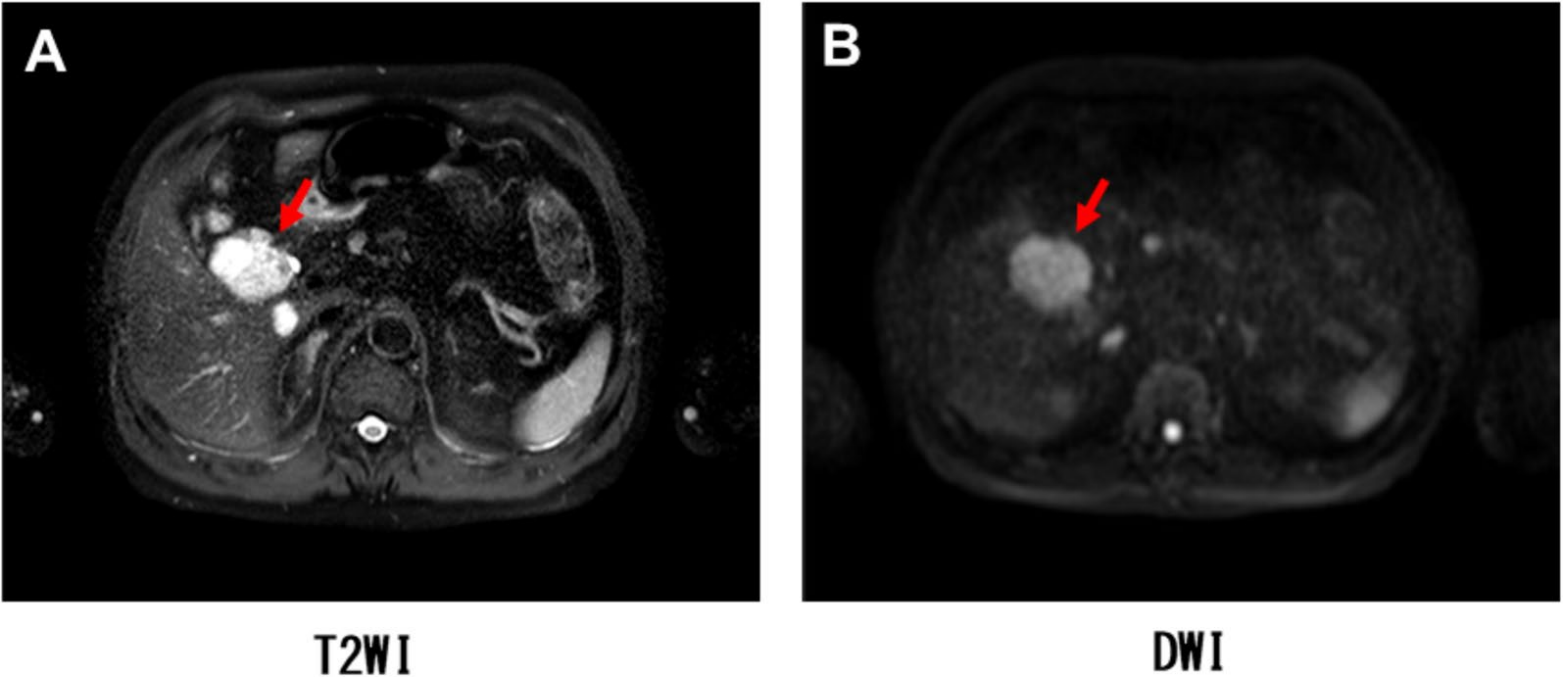
Abdominal magnetic resonance imaging. **A** A mass with a clear border, including hyperintense cystic and reticulated structures on T2-weighted imaging (arrow). **B** A mass with a hyperintense region on diffusion-weighted imaging (arrow)

**Fig. 3:**
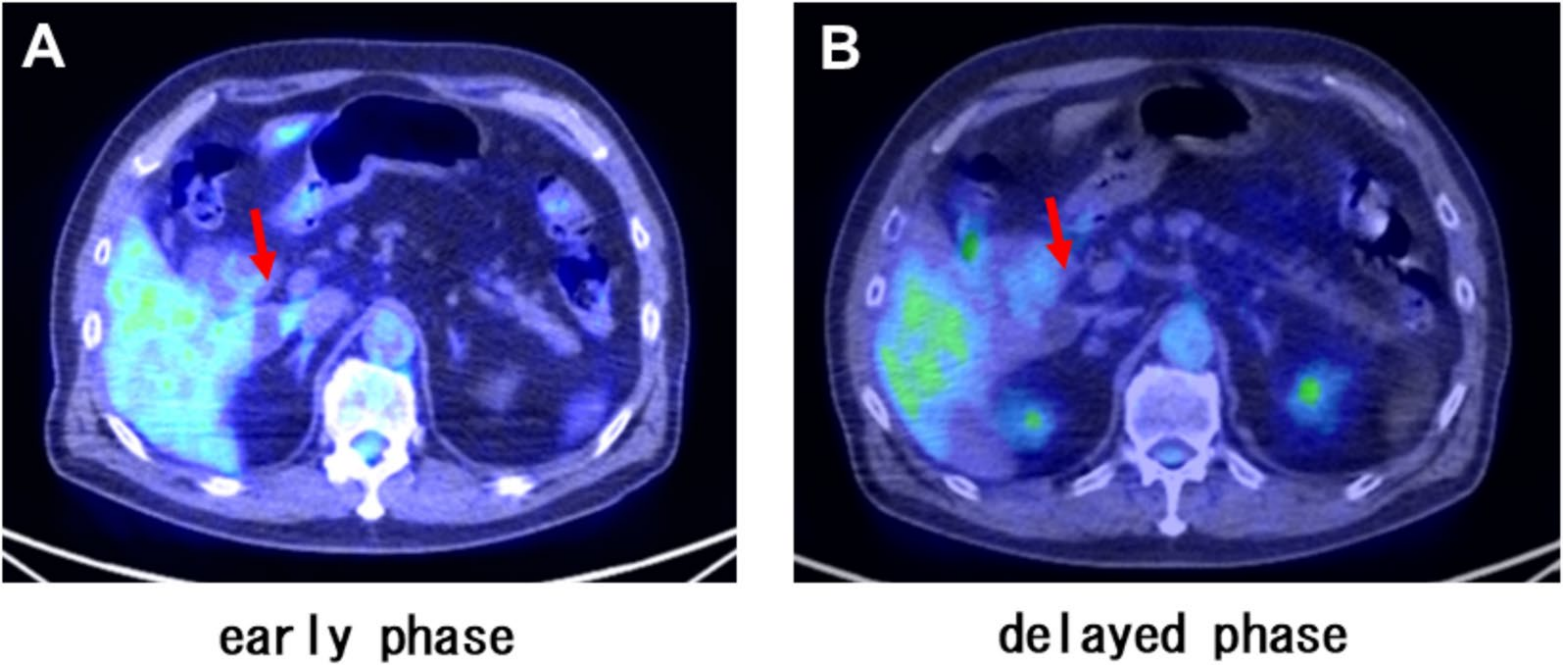
18F-Fluorodeoxyglucose positron emission tomography confirmed high-level FDG uptake in the gallbladder tumor in the early phase (**A**) without increasing FDG uptake in the later phase (**B**)

**Fig. 4:**
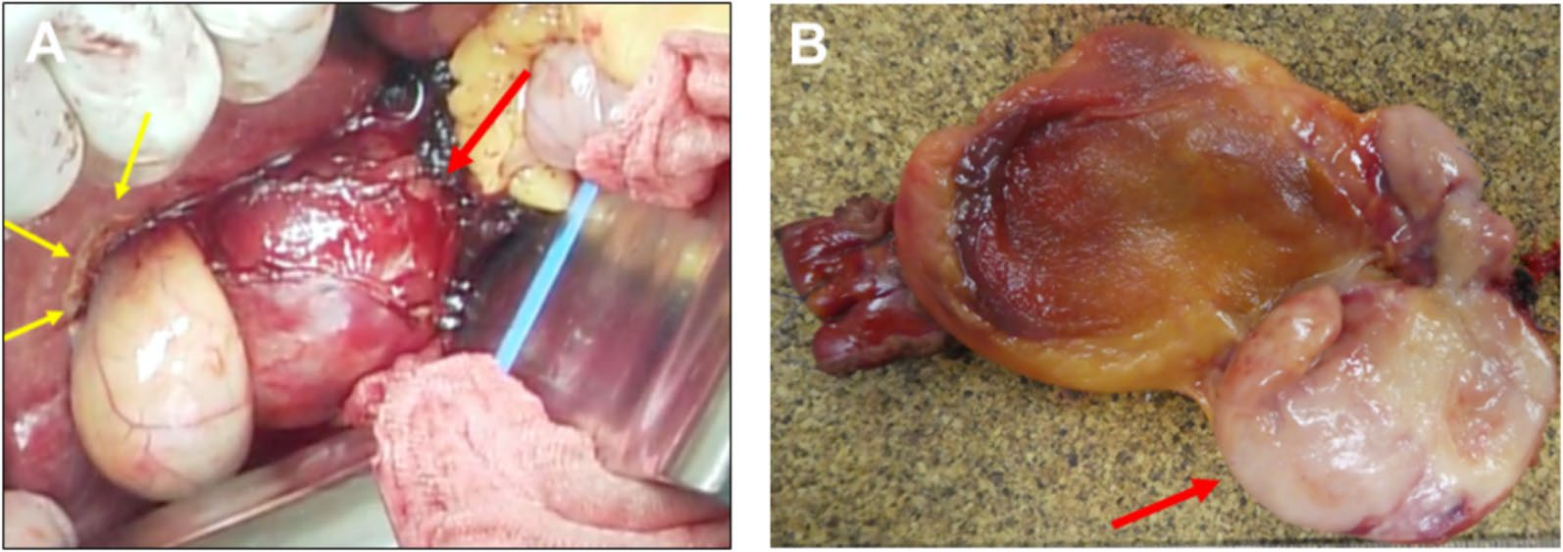
**A** Intraoperative findings showed that the mass was elastic, soft, and slightly poorly mobilized (red arrow). The patient underwent open cholecystectomy with wedge resection (marking line, yellow arrow). **B** In the gallbladder, there was clear-bordered white and elastic soft tumor originating from the gallbladder mucosa (red arrow)

**Fig. 5:**
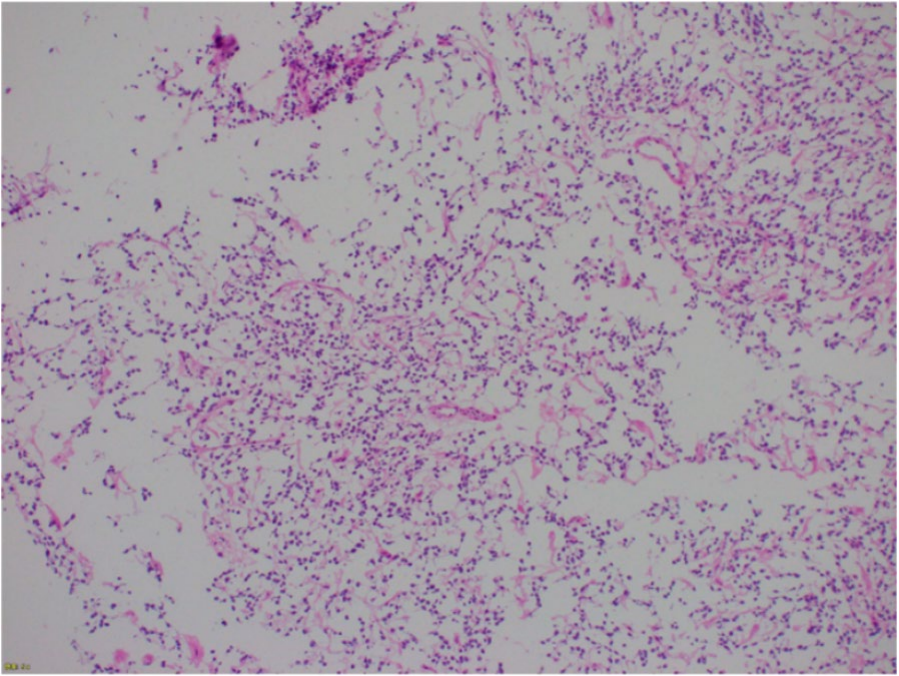
The intraoperative pathological findings show the pools of the mucinous component and variable amount of tumor cells

**Fig. 6:**
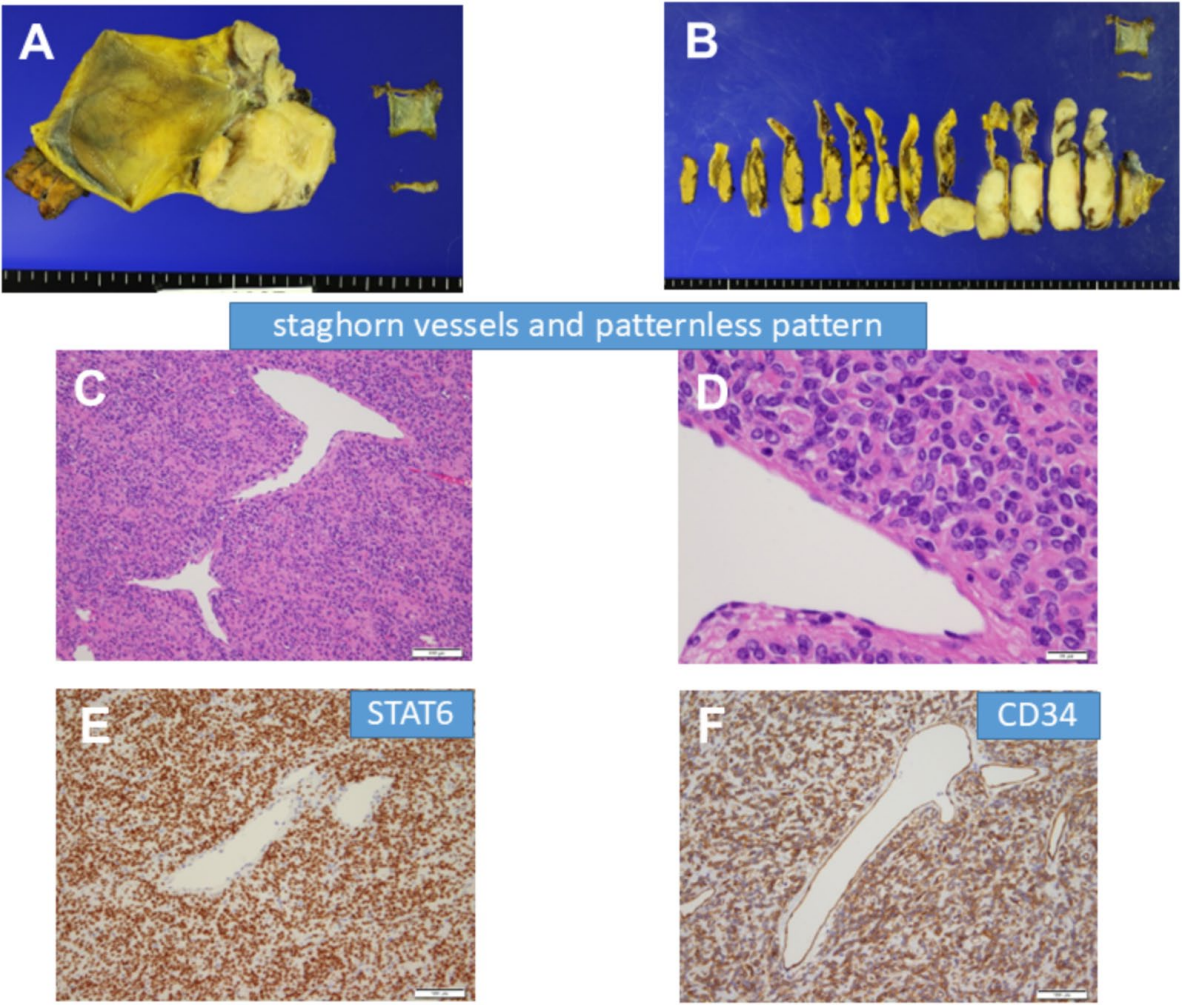
**A**, **B** Resected specimens. **C** Staghorn vessels observed on hematoxylin and eosin (HE) staining (× 40). **D** Patternless growth observed on HE staining (× 200). **E** Immunohistochemical staining for STAT6. **F** Immunohistochemical staining for CD34

A 70-year-old man was admitted for further examination and treatment after abdominal ultrasonography at a clinic revealed a 48-mm tumor in the gallbladder. Laboratory data showed an elevated aspartate transaminase level of 64 U/L, alanine transaminase level of 67 U/L, ICG R15 level of 20%, and normal levels of carcinoembryonic antigen and cancer antigen 19–9 (Table 1). Abdominal ultrasonography revealed a 50-mm hypoechoic mass in the cervix of the gallbladder with no obvious blood flow. Abdominal contrast-enhanced computed tomography (CT) revealed a mass measuring 48 mm in diameter on the right side of the common bile duct (Fig. 1). The border of the mass was clear, with no obvious invasion, and the mass appeared to push out the bile duct from the outside. Abdominal magnetic resonance imaging (MRI) revealed a mass with a clear border that included hyperintense cystic and reticulated structures on T2-weighted images. Diffusion-weighted imaging revealed a hyperintense region of the mass (Fig. 2). 18F-Fluorodeoxyglucose positron emission tomography (FDG-PET) confirmed high-level FDG uptake in the gallbladder tumor in the early phase without increasing FDG uptake in the later phase (Fig. 3). With suspected malignancy, the patient underwent open cholecystectomy with wedge resection of the gallbladder bed. We found that the mass was elastic, soft, and slightly poorly mobilized (Fig. 4). Based on the pathological diagnosis during surgery, the bile duct margin was negative; however, the tumor was strongly suspected to be mucinous carcinoma (Fig. 5). Hence, the patient underwent radical cholecystectomy for gallbladder cancer in addition to extrahepatic bile duct resection, including common bile duct resection, lymphadenectomy of the liver hilum, and choledochojejunostomy. The total amount of bleeding was 1190 mL, and the surgery lasted for 8 h and 19 min because we had to perform an extended surgery. Pathologically, there was no invasion of the common bile duct or gallbladder bed despite the tumor extending into the subserosal fatty tissue surrounding the gallbladder. There was no lymph node metastasis. The tumor showed patternless growth, with vitrified oval- to spindle-shaped tumor cells in the stroma containing both low- and high-density areas of cells. We also observed irregularly dilated vasculature, known as “staghorn pattern”. It was positive for STAT6, CD34, BCL-2, and CD99 but negative for CK(AE1/AE3), C-Kit, ASMA, S100, D2-40, Factor VIII, HMB-45, melan A, and synaptophysin. Ki-67 was 10–15%, and p53 was 5–7% (Fig. 6). Postoperatively, the patient developed an intraperitoneal abscess that was treated using percutaneous drainage. The patient recovered satisfactorily and was discharged on postoperative day 20. The patient was in good general condition without any recurrence at 24 months postoperatively.

**Table 1:** Laboratory data

Blood markers	Index	Normal range
WBC (× 10^3^/μL)	4.26	3.3 – 8.6
RBC (× 10^4^/μL)	465	435 – 555
Hemoglobin (g/dL)	15.2	13.7 – 16.8
Platelets (× 10^4^/μL)	23.2	15.8 – 34.8
ALT (U/L)	64	13 – 30
AST (U/L)	67	10 – 42
ALP (U/L)	220	106 – 322
LDH (U/L)	254	124 – 222
ChE (U/L)	420	240 – 486
γ-GTP (U/L)	60	13 – 64
T-Bil (mg/dL)	0.7	0.4 – 1.5
D-Bil (mg/dL)	0.1	≦0.3
TP (g/dL)	7.5	6.6 – 8.1
ALB (g/dL)	4.5	4.1 – 5.1
BUN (mg/dL)	16.3	8 – 20
CRE (mg/dL)	0.77	0.65 – 1.07
Na (mmol/L)	140	138 – 145
K (mmol/L)	4.6	3.6 – 4.8
Cl (mmol/L)	101	101 – 108
Ca (mg/dL)	10.6	8.8 – 10.1
CRP (mg/dL)	0.1	≦0.14
PT %	116	70 – 140
PT-INR	0.93	0.80 – 1.20
APTT	24.5	23 – 40
ICG R15 (%)	20	≦10
CEA	3.3	≦5
CA19-9	2	≦37

*WBC* white blood cell count, *RBC* red blood cell count, *AST* aspartate aminotransferase, *ALT* alanine aminotransferase, *ALP* aspartate aminotransferase, *LDH* lactate dehydrogenase, *ChE* cholinesterase, *γ-GTP* γ-glutamyl transpeptidase, T-Bil total bilirubin, *D-Bil* direct bilirubin, *TP* total protein, *ALB* albumin, *BUN*, urea nitrogen; *CRE*, creatinine; *CRP*, c-peptide immunoreactivity, *PT* prothrombin time, *PT-INR* prothrombin time-international normalized ratio, *APTT* activated partial thromboplastin time, *CEA* carcinoembryonic antigen, *CA19-9* carbohydrate antigen 19–9, *ICG* indocyanine green

## Discussion

SFTs are a rare mesenchymal neoplasm of fibroblastic differentiation that can occur anywhere in the body. SFTs are composed of spindle-shaped cells that exhibit patternless growth and have both low- and high-density areas of cells. Many cases have been observed in collagenous backgrounds with staghorn-shaped blood vessels [3]. Considering immunohistochemistry, CD34 expression is a consistent finding; however, it can be positive in other types of soft tissue tumors. Leona et al. reported that nuclear expression of STAT6 was positive in almost all SFT cases and was limited to other soft tissue tumors. Strong STAT6 expression has high sensitivity and specificity as an immunohistochemical marker for SFT [4–6].

Since many tumors can have a similar growth pattern, the differential diagnosis is broad. Ostensibly, in cases with dedifferentiation the differential includes mostly malignant neoplasms. Owing to its affinity for the abdomen, gastrointestinal stromal tumor (GIST) should always be considered in the differential diagnosis of dedifferentiated SFT [7]. As with many soft tissue tumors, surgical resection is the standard therapeutic strategy for SFT. Most patients have a good prognosis after surgery, but extra-thoracic SFTs pose relatively higher risks for local recurrence than intrathoracic SFTs [8, 9]. Therefore, complete resection is necessary to reduce the risk of recurrence.

Demicco et al. analyzed 110 cases of SFT and established a 3-tier risk stratification model based on patient age, tumor size, and mitotic figures [10]. They reported that patients over 55 years old, with tumors greater than 15 cm, and mitotic counts greater than 4 mitoses/10 HPFs had the highest risk of metastases and mortality. Our case also showed typical pathological findings of SFT, including patternless growth of spindle cells, staghorn-shaped vessels, and strong nuclear expression of STAT6. There was no evidence of infiltrative growth, necrosis, dedifferentiation, or abnormal mitosis. The tumor had a maximum diameter of 7 cm, and mitotic counts 3 mitoses/10 HPFs. Therefore, the risk factor of our case was only the advanced age of the patient (70 years).

SFTs are rarely observed in the gallbladder, making their identification using preoperative imaging challenging. This complicates the decision-making process for the treatment strategy. When a malignant tumor is suspected in the gallbladder, cholecystectomy or extended cholecystectomy with or without choledochojejunostomy or hepaticojejunostomy is performed, depending on the situation. When it is difficult to make a preoperative diagnosis, pathological diagnosis during surgery is one strategy to avoid oversurgery. The accuracy of pathological diagnosis during surgery for gallbladder tumors is approximately 95% (97.0% for benign tumors and 94.7% for malignant tumors) [11]. From these data, we thought that it is acceptable to use pathological diagnosis as information for deciding the surgical strategy for gallbladder tumors; however, SFT originally have borderline malignant features; therefore, complex interpretations may be required. In fact, the diagnosis during surgery was mucinous carcinoma, although our patient showed typical pathological findings for SFT postoperatively. The background stroma of SFT sometimes shows focal or diffuse mucoid changes [12]. The intraoperative pathological findings show the pools of the mucinous component and variable amount of tumor cells (Fig. 5). Since the findings were already atypical for the gallbladder, they were deemed abnormal and raised suspicion for mucinous carcinoma. Considering the rarity of SFT originating in the gallbladder and the features of pathological findings, including background stroma, background mucoid changes may have influenced the intraoperative pathological diagnosis.

Ginat et al. reported the imaging features of SFT. On CT, an SFT appears as a clearly demarcated, sometimes lobulated, mass with contrast effects. In large tumors, cystic components, calcification, myxoid degeneration, and bleeding may be observed. On MRI, it usually shows low signals on T1- and T2-weighted images; however, if there are many cellular components or if there is edema, necrosis, or myxoid degeneration, it shows high signals on T2-weighted images [13]. In our case, CT revealed a contrast-enhanced mass with clear borders. MRI revealed a mass with a clear border that included hyperintense cystic and reticulated structures on T2-weighted images. Additionally, FDG-PET confirmed high-level FDG uptake in the gallbladder tumor in the early phase without increasing FDG uptake in the later phase. There are no reports on FDG-PET performed for SFTs; this finding may be helpful for imaging findings in future cases of SFTs.

Lazure et al. reported the first case of SFT of the gallbladder [2]. In their case, laparoscopic cholecystectomy was performed because of abdominal pain. Preoperative abdominal ultrasonography revealed cholelithiasis and thickening of the fundus of the gallbladder without a distinctive mass. Thus, the tumor was incidentally detected in the resected specimen. Therefore, this is the first report of an SFT of the gallbladder that was detected preoperatively.

## Conclusions

We report a rare case of a primary SFT originating in the gallbladder. Clinicians should be aware that SFT can be found in the gallbladder, and when it is difficult to make a preoperative diagnosis, surgical treatment should be considered.

## Data Availability

All data generated or analyzed during this study are included in this published article.

## Abbreviations

SFT: Solitary fibrous tumors
CT: Computed tomography
MRI: Magnetic resonance imaging
FDG-PET: 18F-Fluorodeoxyglucose-positron emission tomography
